# Anti-Apoptotic Effects of Osteopontin via the Up-Regulation of AKT/mTOR/β-Catenin Loop in Acute Myeloid Leukemia Cells

**Published:** 2017-04-01

**Authors:** Mahdi Zahed Panah, Mohsen Nikbakht, Seyed Mehdi Sajjadi, Shahrbano Rostami, Amir Hossein Norooznezhad, Hosein Kamranzadeh Fumani, Ardeshir Ghavamzadeh, Saeed Mohammadi

**Affiliations:** 1 Department of Medical Laboratory Sciences, Faculty of Allied Medicine, Qazvin University of Medical Sciences, Qazvin, Iran; 2 Hematology-Oncology and Stem Cell Transplantation Research Center, Tehran University of Medical Sciences, Tehran, Iran; 3 Cellular and Molecular Research Center, Birjand University of Medical Sciences, Birjand, Iran; 4 Medical Biology Research Center, Kermanshah University of Medical Sciences, Kermanshah, Iran

**Keywords:** Curcumin, Acute myeloid leukemia, Osteopontin

## Abstract

**Background: **The conventional chemotherapeutic regimens which applied for treatment of acute myeloid leukemia (AML) mostly target tumor bulk but not leukemic stem cells (LSCs). Aberrant expression or activation of mediators such as osteopontin (OPN) or PI3K/PTEN/Akt/mTOR pathway plays a key role in making prone to develop leukemia. Preventing or treating cancer by curcumin (CUR) has been suggested recently. CUR induces apoptosis and growth inhibition through various mechanisms in leukemic cells. In present study, we tried to measure the toxic response in vitro to CUR for evaluation ofchangesin cell viability, survival and molecular-mediated resistance in primary AML cells.

**Materials and Methods:** Isolated primary CD34+/CD38− bone marrow derived AML cells were treated with CUR, Daunorubicin (DNR) and/or their combination by MTT assay, Annexin V/PI staining, and colony-formation. The mRNA expression of OPN/AKT/mTOR/PTEN/β-catenin genes was measured by Real-Time PCR. The siRNA against OPN was applied for CUR- treated cells.

**Results: **Growth inhibition effect of DNR increased in combination with CUR on primary CD34+/CD38- AML cells. Suppression of OPN with siRNA increased the cytotoxic effects of CUR. Likewise, OPN gene expression increased in response to CUR treatment in AML cells. AKT, mTOR, β-catenin or PTEN gene expression increased by CUR, but OPN siRNA decreased the level of mRNA expression of mentioned molecular pathway.

**Conclusion** **:** The chemo-resistance of AML cells against therapy might be relevant to increasing of OPN mRNA expression and activity of other mediators including AKT, mTOR, PTEN, and β-catenin. In this context, targeting of OPN might be more impact on CD34+ AML cells.

## Introduction

Acute myeloid leukemia (AML) is a clonal disorder through transformation and uncontrolled proliferation myeloid progenitor cells. The conventional chemotherapeutic regimens used for induction of complete remission (CR) consist of the combination cytarabine and an anthracycline such as DNR.^1^^,^^2^ These therapies mostly target leukemic bulk but not leukemic stem cells (LSCs).^3^ LSCs phenotype has been described as CD34+/CD38- and can arise from both normal hematopoietic stem cells and differentiated hematopoietic progenitor cells.^4^^,^^5^

LSCs are rare subpopulation which initiating a leukemogenic state and could be the factor of the recurrence and cause a problem in development of the curative therapies. LSCs may be affected by initiating events causing the loss of ability of cells to differentiation, but retain the ability to self-replication, proliferation, and resistance to apoptosis. ^1^^,^^6^ Aberrant expression or activation of mediators in PI3K/PTEN/Akt/mTOR pathwayas, plays a key role in making prone to develop leukemia.^7^ Various cytokines such as osteopontin (OPN) can exert their effects on cells through this pathway.^8^

Osteopontin (OPN) is a glycoprotein expressed by cells in a variety of tissues. OPN molecules are preserving cell viability in response to anticancer agents which its receptors could be purposed as a therapeutic targeting of cancer cells^9^^, ^^10^. There are two different forms of OPN as secreted (sOPN) and intracellular (iOPN) protein. Many integrins such as αvβ3 as well as CD44 are able to stimulate OPN signal transduction in cells.^11^Some purposed mechanisms of OPN are available regarding to the apoptosis blocking in endothelial cells and implication in the cell survival through Akt pathway.^11^^, ^^12^

Recent study in the regulation of OPN expression in AML showed that high basal Akt phosphorylation, activated form, results in a significant decrease in OPN mRNA expression. OPN stimulation is not able to induce significant Akt phosphorylation.^13^The upregulation of OPN has been described in poor-prognosis patients with AML. The knockdown of OPN expression induces cell death in AML blasts, CD34+/CD38-/CD123+ leukemic stem and also progenitor cells (LSPCs).^13^ Higher levels of marrow OPN in AML patients implies the prognostic factor role for OPN compared to normal control patients.^14^ The prominent efforts for therapy in AML are being directed toward identifying therapeutic targets to eradicate quiescent leukemia-initiating cells (LICs) without any impact on normal hematopoiesis. Dramatic advances in targeted therapy have been dependent on fundamental understanding of molecular pathways involved in progression of the leukemia and finding a compound that blocks these pathways. Thus, interfering with the cell proliferation is a critical role for antineoplastic drugs leading to cell death.

CUR is isolated from the rhizome of curcuma longa and gives the yellow color to turmeric. Preventing or treating cancer by CUR has been suggested recently.^15^ CUR induces apoptosis and growth inhibition through various mechanisms in tumor cells.^16^ Involving of the BCL-2 in AML cells during CUR treatment is associated with apoptosis^17^^,^^18^ . In the present study, we tried to measure the toxic response in vitro to CUR to evaluate changes in cell viability, survival and molecular-mediated resistance in primary CD34+/CD38- AML cells.

## MATERIALS AND METHODS

**Materials**

CUR was purchased from Sigma-Aldrich and dissolved in dimethyl sulfoxide (DMSO) as a stock solution of 100 mM and stored at -20°C. DNR (Pharmacia & Upjohn SpA; Milan, Italy) was dissolved in distilled water to prepare 1 mg/ml stock solution and 100 µg/ml working solution immediately before use. Annexin V-Alexa Fluor-488/PI kit was purchased from BD Biosciences (San Jose, CA, USA). The human monoclonal antibodies PE anti-CD34 and FITC anti-CD38 were purchased from BD Biosciences (San Jose, CA, USA).CD34 Multi Sort Micro Bead kit was obtained from Miltenyibiotec Inc (Miltenyibiotec Inc, Auburn, CA). Tripure isolation reagent was purchased from Roche Applied Science (Germany). The cDNA synthesis kit and SYBR ® Premix Ex Taq™ were purchased from Takara Biotechnology Co (Otsu, Japan).

Cell Culture

Bone marrow (BM) aspirates were obtained from 10 newly diagnosed AML patients prior treatment. All patients provided written informed consent. The study was approved in the Ethics Committee at the Hematology-Oncology and Stem Cell Research Center(ir.tums.horsct.rec.1394.103.5).Bone marrow mononuclear cells (BMNCs) were isolated by Ficoll-Hypaque density gradient centrifugation method.TheCD34+/CD38− cells were enriched by using Multi Sort CD34 MACS Column Technology. The separated cells were stained with PE-conjugated anti-CD34 and FITC anti-CD38 to determine the purity of CD34+ cells. RPMI 1640 supplemented with 10% FBS (Gibco; Invitrogen, USA) and 2 mM L-glutamine, 100 units/mL penicillin and 100 μg/mL streptomycin was used for primary culture. Cells were incubated at 37°C in a humidified atmosphere containing 5% CO2.

**MTT Assay**

Cells cultured in triplicated at 5×10^3^/100µl cell density in 96-well culture plates (SPL Life sciences, Pocheon, Korea) treated with different concentrations of CUR(20, 40, 80 μM) and 0.5 μg/ml of DNR and/or their combination with 40μM of CUR for 24 hours at 37° C in a humidified atmosphere containing 5% CO2. Then, the cells were incubated for 4 hours with 3-(4,5-dimethylthiazol-2-yl)-2,5-diphenyltetrazoliumbromide (MTT, 5 mg dissolved in 1ml of PBS, Sigma, St. Louis, MO, USA).The plates were spun, and the purple formazan crystals of metabolized yellow tetrazolium salt by viable cells were dissolved in DMSO. Absorbance was quantified at 570 nm using the ELISA plate reader (Micro plate Reader; Bio-Rad). Results were expressed as a percentage of proliferation with 100% representing control cells treated with 0.1% DMSO alone.

**Evaluation of Apoptosis by Annexin V/PropidiumIodide (PI) Assay**

A density of 1×10^6^/ml cells per well in 6-well plates was treated with CUR, DNR and also their combination in indicated concentrations. After 24 hours, cells were harvested and mixed with Annexin V-Alexa Fluor-488/PI according to the manufacturer’s introduction. The stained cells were examined by flow cytometry (Partec, Munich, Germany). Discrimination of cells was performed as apoptosis (Annexin V^+^/PI^-^ [early apoptosis], Annexin V^+^/PI^+^ [late apoptosis]) and necrosis (Annexin V^-^/PI^+^).

**Colony Forming Assay**

AML cells were suspended at a density of 2000 cells in 0.5 ml of RPMI 1640 mediums, and then treated with CUR alone and OPN siRNA. The treated and untreated cells plated in Methocult semi solid media (Stem Cell Technologies, Vancouver, BC, Canada). After an incubation period of 14–16 days,the colonies were enumerated by inverted microscope. Accumulation of ≥50 cells were scored as granulocyte-macrophage colony-forming units (GM-CFU) and collection of 3~50 cells were considered as one cluster. Three independent experiments were performed.

**Real-Time PCR**

Total RNA of the DNR, CUR or the combination of treated and untreated isolated cells was extracted with Tripure isolation Reagent according to the manufacturer’s instruction. Complementary DNA (cDNA) was reverse transcribed by using cDNA synthesis kit (Takara Bio Inc., Otsu, Japan). Real-Time PCR was performed with Step One Plus™ (Applied Biosystems, CA, USA) using SYBR Premix Ex Taq technology (Takara Bio Inc., Otsu, Japan). Data were normalized to HPRT expression in each sample. Analysis of relative gene expression data was performed using the 2^-∆∆Ct^ method. Table 1 shows the primer sequences for genes used.

**Table 1:** The characters of the used primers in Real-Time PCR

**Gene**	**Forward Primer (5'-3')**	**Reverse Primer (5'-3')**	**Size ** **(bp)**
**HPRT**	TGGACAGGACTGAACGTCTTG	CCAGCAGGTCAGCAAAGAATTT A	111
**OPN**	ACCCTTCCAAGTAAGTCCAAC G	GGTGAGAATCATCAGTGTCATC TAC	139
**AKT1**	AGCGACGTGGCTATTGTGAAG	GTACTCCCCTCGTTTGTGCAG	51
**mTOR**	AACTCCGAGAGATGAGTCAAG A	AGTTGGTCATAGAAGCGAGTAG A	49
**PTEN**	TGGATTCGACTTAGACTTGAC CT	TTTGGCGGTGTCATAATGTCTT	184
**β-** **Catenin**	TACCTCCCAAGTCCTGTATGA G	TGAGCAGCATCAAACTGTGTAG	180

**Short Interfering RNA (siRNA) Transfection**

The siRNA against OPN was applied to CUR- treated cells using lipofectamin2000 reagent (LF2000; 10µg/ml; Invitrogen) according to the manufacturer’s instruction. Cells were lysed 24 h post-transfection and quantitative Real-Time PCR was performed using following sequences of OPN siRNA primers: 5´-GGAAUAUUACUGUGGGAAAdTdT -3´ (sense) and 5´-UUUCCCACAGUAAUAUUCCdTdT-3´ (anti-sense). Highest transfection efficiency was obtained in the experiments within 24 hours post-transfection at a final concentration of 40(Pm).This optimal condition was used for subsequent experiments tested with siRNA.

**Flow Cytometry**

Isolated cells were treated with CUR, DNR and their combination within 24 hours. Before labeling, the cells were spun to remove the debris and re-suspended in PBS. Then, the cells were stained with the panel of human monoclonal antibodies including PE anti-CD34, FITC anti-CD38. The analysiswas carried out by a Partec PAS III flow cytometer (Partec, Munich, Germany), and data were interpreted using the FloMax software.

**Statistical Analysis**

Using IBM SPSS Statistics 19 software, grouped data were presented the groups of data was presented as means ± S.E and were compared by One-way analysis variance (ANOVA).

## Results

**Growth Inhibition Effect of DNR Increases in Combination with Curcumin on Primary AML Cells**

**Figure 1:**
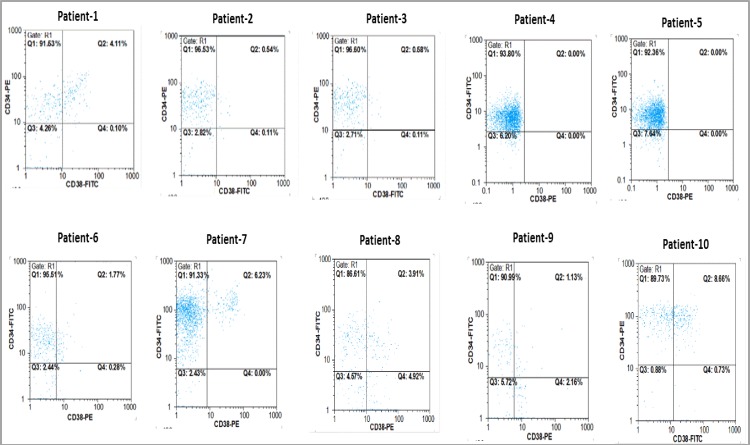
Primary CD34+/CD38- cells isolated from BMMCs of 10 AML patients were isolated and subjected to flow cytometry to determine the purity of CD34+/CD38- cells.

The growth inhibitory effects of CUR and DNR were evaluated on primary CD34+/CD38- BM derived. The purity of isolated cells was more than 92% in most cases (Figure 1).

Dose-response curves with different concentrations of CUR using MTT assay at 24hours showed that the cell viability in comparison with untreated cells significantly decreased to 79% at 40 µM and 70% at 80µM in primary AML cells, respectively. We could not estimate the IC50 value because of the presence of a significant percentage of residual leukemic cells.

**Figure 2:**
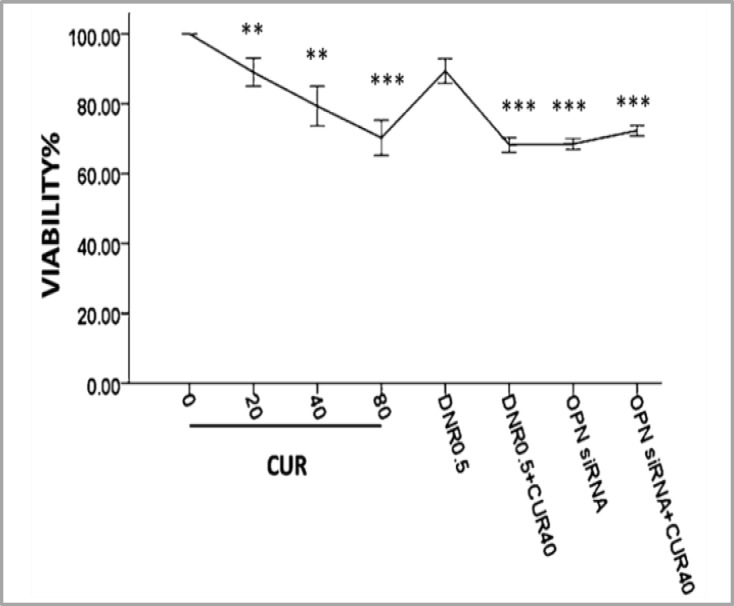
Dose-response curves with different concentrations of curcumin (µM) and daunorubicin 0.5µg/ml using MTT assay/24h showed that growth inhibition effect of daunorubicin increases in combination with curcumin on primary AML cells. OPN siRNA (40pmol/ml) addition or subsequent treatment with curcumin (40µM) decreased the cell viability. The graphs represent three independent experiments for all patients (mean ± S.E). *P<0.05, **p<0.01, ***p<0.001 (compared with control or comparisons depicted).

**Figure 3:**
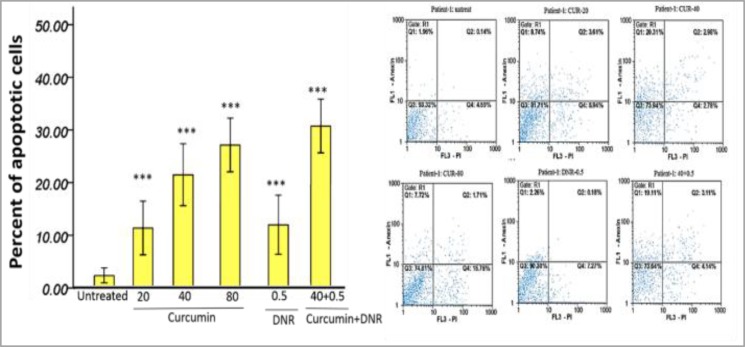
The performance of the Annexin-V/PI staining on treated cells with curcumin (µM) and daunorubicin 0.5µg/ml. The graphs represent three independent experiments for all patients (mean ± S.E). *P<0.05, **p<0.01, ***p<0.001 (compared with control).

Overall, the cell viability alteration after treatment with DNR alone was significantly less than CUR. Meanwhile, the treatment of the cells with the combination of CUR and DNR significantly increased the lethality of CUR alone with the same dose (40µM). OPN siRNA (40pmol/ml) addition or subsequent treatment with CUR (40µM) decreased the cell viability (Figure 2 and supplementary Figures 1-2). Annexin-V/PI staining indicated that both CUR and DNR-inhibited cell growth were induced by apoptosis (Figure 3 and supplementary Figure 3).

**Suppression of OPN with siRNA increased the Cytotoxic Effects of Curcumin**

**Figure 4:**
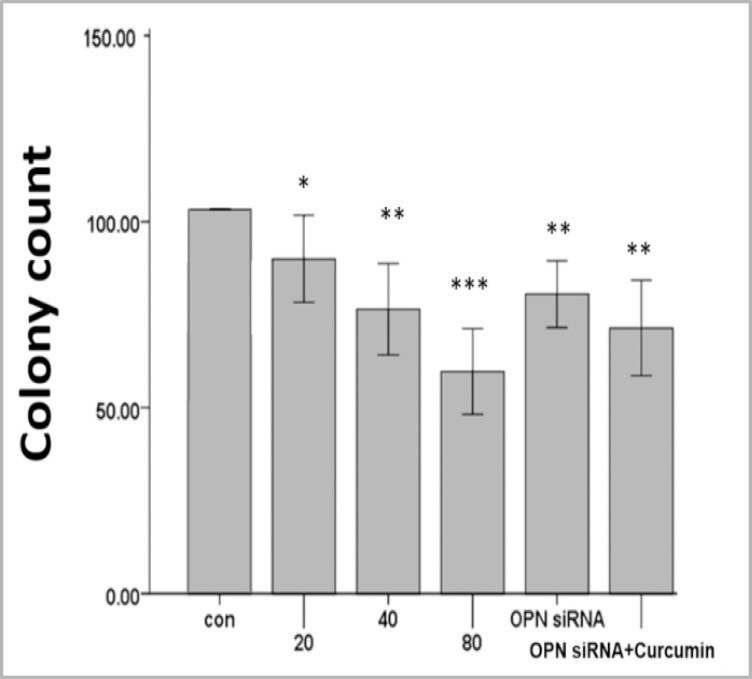
Treatment with various concentrations of curcumin as well as suppression of OPN with optimized siRNA (40pmol/ml) and subsequent treatment with curcumin for 24h decreased the clonogenic growth compared with the control in primary AML cells. Three independent experiments performed for all patients (mean ± S.E). *P<0.05, **p<0.01, ***p<0.001

We used OPN-specific siRNA to block the OPN function and investigated the effect of the OPN-mediated enhancement of AML cells on survival and sensitivity to CUR. OPN siRNA transfection alone or subsequent treatment with CUR decreased the clonogenic growth compared with the control group in primary AML cells (Figure 4 and supplementary Figure 4).

In accordance with this result, the cell viability decreased when OPN gene was inhibited (Figure 2 and supplementary Figure 1-2). These observations imply that OPN plays an important role in the regulation of survival and proliferation of AML cells.

**OPN Gene Expression Increased by Curcumin in AML Cells**

**Figure 5:**
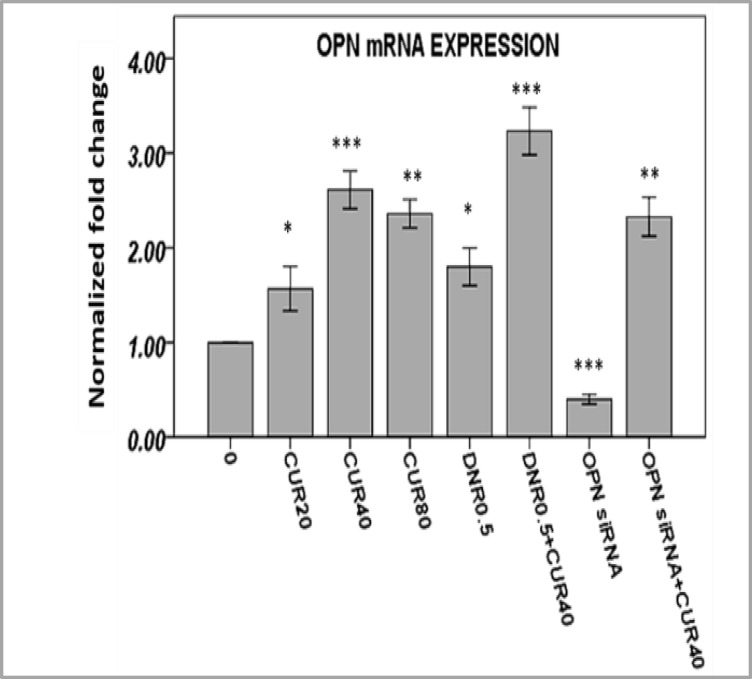
Treatment of AML cells with curcumin and daunorubicinfor 24h showed that OPN gene expression was significantly strengthened using Real Time-PCR. Three independent experiments performed for all patients (mean ± S.E). *P<0.05, **p<0.01, ***p<0.001.

To determine the effects of CUR on transcriptional expression of OPN in response to CURand/or DNR, we investigated mRNA levels of OPN by quantitative RT-PCR. OPN gene expressions were significantly strengthened by different concentrations of CUR in AML cells (Figure 5 and supplementary Figure 5-6). The over-expression of OPN mRNA levels might imply an exploitation of OPN to apoptosis resistance by these cells.

**AKT, mTOR, β-catenin or PTEN Gene Expression Increased by Curcumin but Decreased by OPN siRNA**

**Figure 6:**
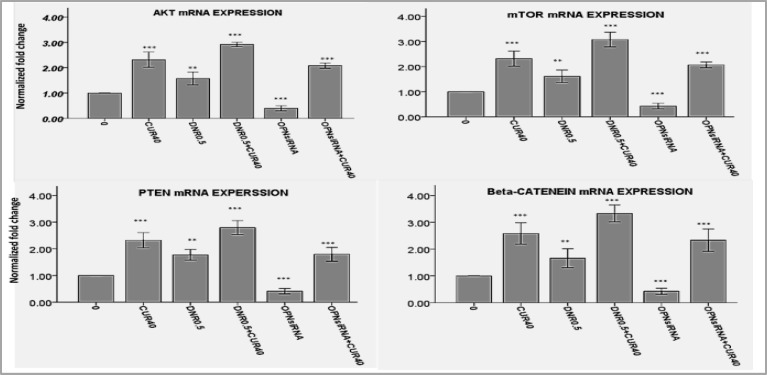
The examination of the effects of curcumin or OPN suppression by siRNA on transcription of AKT1, mTOR, β-catenin and PTEN genes, relative to HPRT, by Real Time-PCR showed that expression of these genes increased by curcumin, but decreased by OPN siRNA. Three independent experiments performed for all patients (mean± S.E). *P<0.05, **p<0.01, ***p<0.001.

Similar to OPN gene expression, some crucial regulators in cell survival such as AKT, mTOR, β-catenin and PTEN increased when AML cells were treated with CUR or DNR. The gene expression of these molecules reduced when cells were treated with OPN siRNA, but they increased by more than twofold as compared to control group in response to treatment with CUR (Figure 6A-D and supplementary Figures 7-8).Based on these findings, it appears that OPN might control the expression of mentioned genes at the transcription level, but CUR is a stimulus strong enough to compensate for OPN lack or scarcity.

## Discussion

 Our data indicated that CUR induced apoptosis and reduced colonization potency in a fraction of CD34+/CD38-AML cells. The existence of a significant percentage of viable cells, led us to deduce that these cells could be relatively refractory to CUR.Moreover, the crucial components of cell growth regulatory genes such as OPN, AKT, mTOR, β-catenin or PTEN elevated in response to CUR and DNR treatment. These genes are oncogenes or tumor suppressor that have important role in the control of the cell cycle or apoptosis and they are frequently activated or suppressed in AML.^19^However, CUR was more toxic for these cells than DNR. Meanwhile, OPN inhibition exerted an anti-survival effect on CD34+/CD38- AML cells. Primitive human LSCs defined by CD34+ populations which can be nonresponsive to conventional chemotherapy and bring about minimal residual disease and relapse.^5^^,^^20^^,^^21^

Resistance of CD34+ AML cells to DNR has been displayed in CD34+ cells KG-1a and KG-1.^22^ Resistance to DNR may not only come from P-gp-mediated efflux but also due to its distribution feature in CD34^+^cells.^23^^, ^^24^In addition, PI3K pathway activity can play role in the protection of cell against DNR.^25^ Actually, survival signaling and evasion process of apoptosis should be disrupted with a drug to beat down cell resistance.

Herbal therapy as a complementary and alternative medicine has been proposed to find an anti-neoplastic agent with the most toxicity on AML cells and the least toxic effects on normal cells. CUR selectively induces apoptosis and kills tumor cells by modulating different molecular pathways.^16^Although we found no significant difference in apoptotic rate of AML cells between combined treatment of DNR and CUR or CUR alone, CUR could increase DNR efficacy in cell death. Meanwhile, this combination was also accompanied with the high expression of all studied genes.

The pro-apoptotic and growth inhibition potency of CUR in cancer cells are expressed in critical mechanisms interferes such as directly or indirectly control of multiple genes expression or molecular activation.^16^ In line with our result , the current study showed that some cancer cells such as human gastric cancer cells can display resistance to CUR through serving pro-survival mediators, including OPN or PI3K/AKT.^26^In another study of CUR effects on AML CD34^+^ KG-1 and CD34^-^ U937 cell lines, increased mRNA levels of OPN/AKT/mTOR/PTEN/β-catenin have been observed.^27^Nevertheless, in frequent surveys CUR have could inhibit phosphorylation, activated form, of AKT/mTOR in various cancer cells.^28^^,^^29^

Also, the study on the effect of CUR on β-catenin in human neuroblastoma cell line SH-SY5Y has shown an apparent increase in the expressionof mRNA and protein level of β-catenin and PI3K/AKT.^30^^,^^31^Similarly, CUR by activation of GSK-3βmay reduce the expression of β-catenin and its downstream target cyclin D1 in medulloblastoma. Given that, GS3K-inhibited cells have shown increased β-catenin levels,^30^ on the other hand, AKT and mTOR inactive GS3K. Based on these result, CUR might exert its effect on β-catenin through overexpression of AKT and mTOR.^32^

In our study, OPN-specific siRNA either alone or together with CUR could decrease the viability, colonogenesis of AML cells and mRNA levels of AKT, mTOR, PTEN, and β-catenin gene axis. However, OPN-siRNA transfected cells tried to increase cited gene axis after treatment with CUR. High level of OPN expression effects on tumor genesis and cell protection from cytotoxic agents has been presented.^11^ Meanwhile, co-expression of OPN receptor CD44 with CD123 on CD34+/CD38- AML cells could confirm that OPN is a key regulator in LSCs.^33^ Various oncogene molecules, including PI3K/AKT, Wnt-β-catenin, and P70S6K/mTOR can be up-regulated by OPN that results in tumor growth or apoptosis inhibition as a cytoprotective action. ^10^^, ^^34^^-^^37^ Reciprocally, some oncogenes, including BCR-ABL-induced OPN over-expression via involving a number of signaling molecules such as PI-3K.^38^^, ^^39^

PI3K/AKT pathway is one of the multiple pathways by which OPN through integrin can emerge in the regulation of cancer cells. It has also been reported that mRNA isoform (OPN-c) significantly activates ovarian cancer cell proliferation and anchorage-independent growth through PI3K/Akt pathway. The levels of PTEN expression might be coincided with the activation of Akt and OPN.^40^^, ^^41^

Matsuura et al. demonstrated that over expression of OPN in ovarian clear cell carcinoma induced extra cellular matrix (ECM) invasion in vitro. Furthermore, they showed that down-regulation of OPN in response to simvastatin treatment, and also transfection with OPN-specific siRNA reduced cell invasiveness.^40^

Mason et al. demonstrated that high levels of OPN and β-catenin expression in various cancer cells can establish a poor patient survival rate. The inhibition effects of Agelastatin as a natural component on OPN expression might reduce cell invasiveness and subsequently increase survival rate.^41^

## CONCLUSION

 Based on these results, it could be concluded that although CUR improved the cell viability inhibition of DNR, many cells still give a protection with increasing expression of crucial mediators that bestow a resistance apoptosis advantage upon the CUR-treated cells. This chemo-resistance might be relevant to increasing of OPN expression contributing to expression or activity of other mediators including AKT, mTOR, PTEN, and β-catenin. As a consequence, abrogation of pro-survival mediators could sensitize cancer cells to CUR. In this context, targeting of OPN might be more likely to impact the CD34+ AML cells as an insensitive cell to chemotherapy. 

## SUPPLEMENTARY FILES

SUPPLEMENTARY FILES AMLOPNSF
