# Supplementary material for: Anti-Apoptotic Effects of Osteopontin via the Up-Regulation of AKT/mTOR/β-Catenin Loop in Acute Myeloid Leukemia Cells
Source: Int J Hematol Oncol Stem Cell Res. 2017 Apr 1;11(2):148–57. (PMC5574411)
Supplement: SUPPLEMENTARY FILES — AMLOPNSF [file IJHOSCR-11-148-s001.zip › Suplementary figures.docx]

**Supplementary figures**


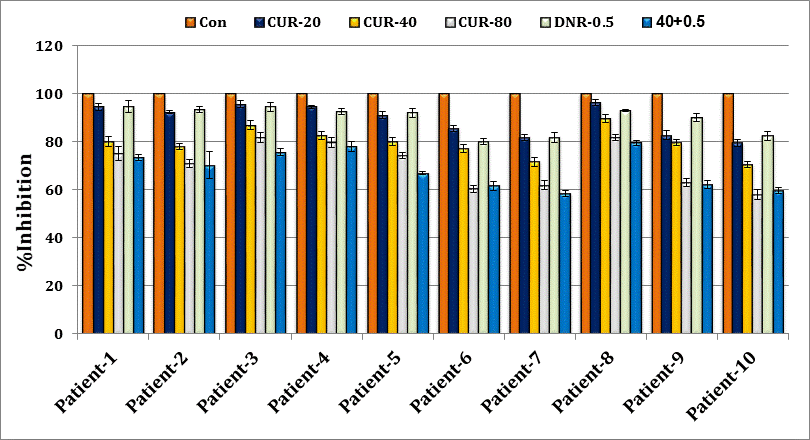


**Supplementary figure 1:** Effects of curcumin (0-80 µM) and danarubicin (0.5µg/ml) treatment on cell viability of primary CD34+/CD38- AML sample. Data are mean ± S.E of three independent experiments.


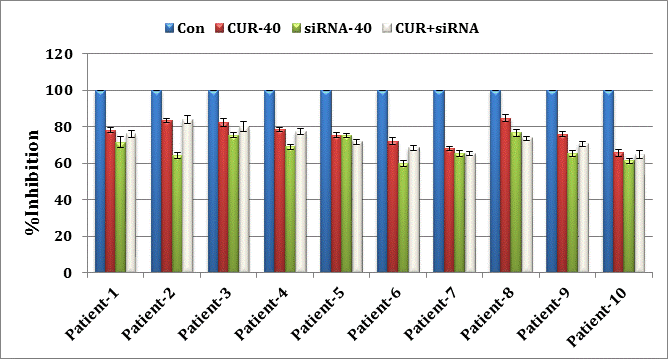


**Supplementary figure 2:** Effects of curcumin (40 µM), siRNA (40pol/ml) and curcumin (40 µM) +siRNA (40pmol/ml) treatment on cell viability of primary CD34+/CD38- AML sample. Data are mean ± S.E of three independent experiments.


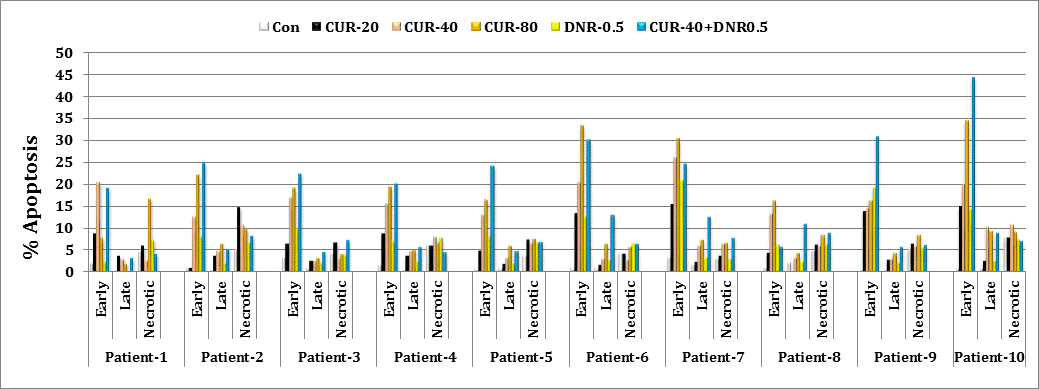


**Supplementary figure 3:** The performance of the Annexin-V/PI staining on treated of primary CD34+/CD38- AML sample with curcumin (0-80µM) , danarubicin (0.5µg/ml) and curcumin (40µM) +daunorubicin (0.5µg/ml)

.


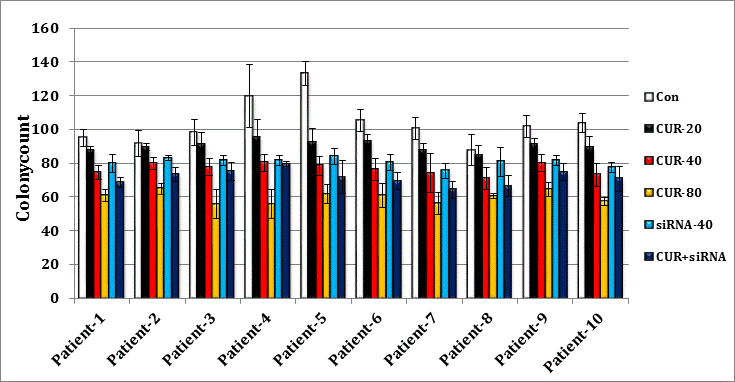


**Supplementary figure 4**: Treatment with various concentrations of curcumin as well as suppression of *OPN* with optimized siRNA (40pmol/ml) and subsequent treatment with curcumin for 24h decreased the clonogenic growth compared with the control in primary CD34+/CD38-AML cells. Data are mean ± S.E of three independent experiments

.
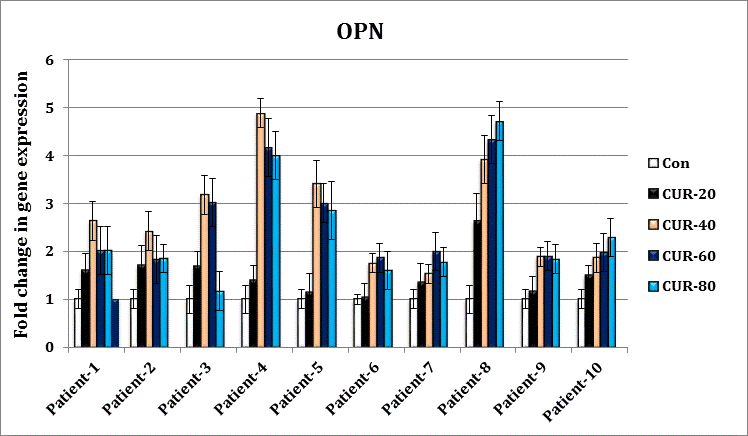


**Supplementary figure 5 :** Effects of curcumin (0-80µM) treatment on *OPN* gene expression of patient samples were evaluated by Real time PCR**.** Data are mean ± S.E of three independent experiments.


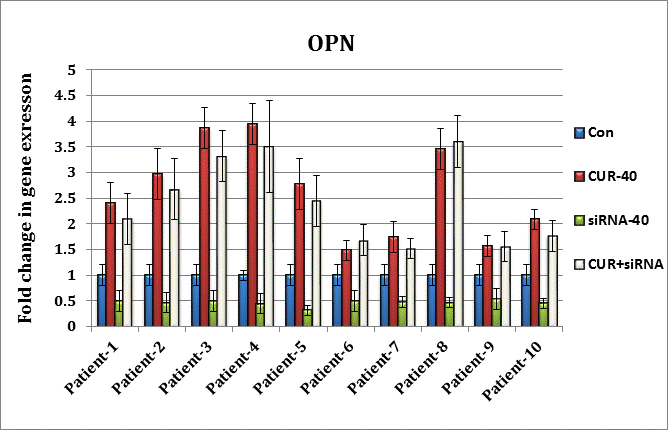


**Supplementary figure 6 :** Effects of OPN specific siRNA (40 pM) transfection and curcumin (40µM) treatment on *OPN* gene expression was evaluated by Real time PCR in all patients samples**.** Data are mean ± S.E of three independent experiments


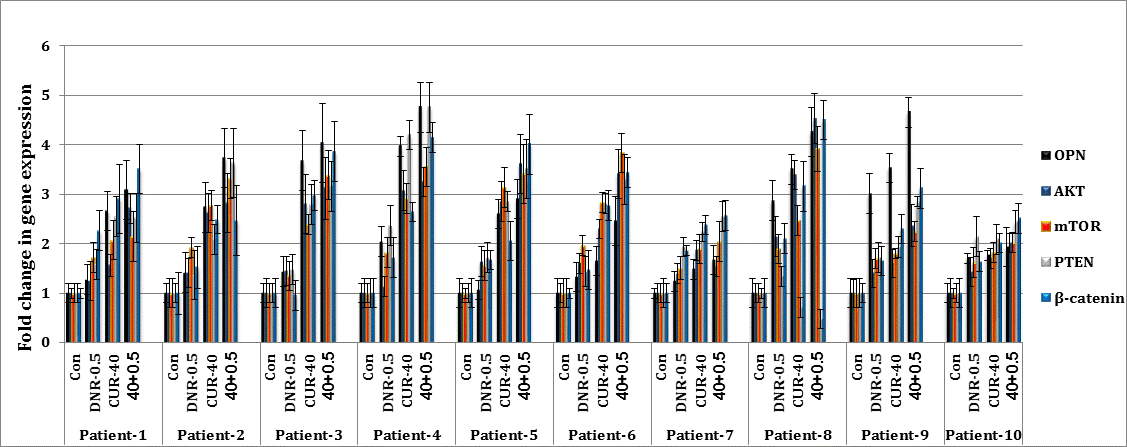


**Supplementary figure 7**: Effects of curcumin (40µM) and daunorubicin (0.5µg/ml) and their combination treatment on AKT/mTOR/PTEN/β-catenin genes were evaluated by Real time PCR**.** Data are mean ± S.E of three independent experiments


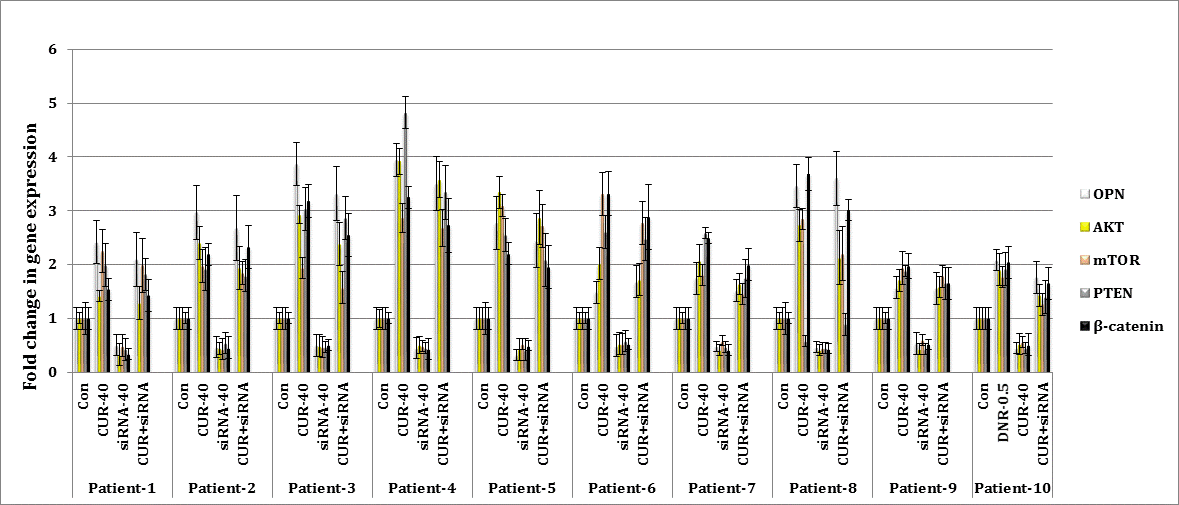


**Supplementary figure 8**: Effects of curcumin (40µM), siRNA (40 pM) and their combination on AKT/mTOR/PTEN/β-catenin genes were evaluated by Real time PCR**.** Data are mean ± S.E of three independent experiments
